# *GhMPK16*, a novel stress-responsive group D MAPK gene from cotton, is involved in disease resistance and drought sensitivity

**DOI:** 10.1186/1471-2199-12-22

**Published:** 2011-05-16

**Authors:** Jing Shi, Liang Zhang, Hailong An, Changai Wu, Xingqi Guo

**Affiliations:** 1 State Key Laboratory of Crop Biology, Shandong Key Laboratory of Crop Biology, Shandong Agricultural University, Taian, Shandong 271018, China

**Keywords:** Cotton (*Gossypium hirsutum*), *GhMPK16*, Pathogen resistance, Drought sensitivity

## Abstract

**Background:**

Mitogen-activated protein kinase (MAPK) cascades play pivotal roles in mediating biotic and abiotic stress responses. In plants, MAPKs are classified into four major groups (A-D) according to their sequence homology and conserved phosphorylation motifs. Members of group A and B have been extensively characterized, but little information on the group D MAPKs has been reported.

**Results:**

In this study, we isolated and characterised *GhMPK16*, the first group D MAPK gene found in cotton. Southern blot analysis suggests *GhMPK16 *is single copy in the cotton genome, and RNA blot analysis indicates that *GhMPK16 *transcripts accumulate following pathogen infection and treatment with multiple defense-related signal molecules. The analysis of the promoter region of *GhMPK16 *revealed a group of putative *cis*-acting elements related to stress responses. Subcellular localization analysis suggests that GhMPK16 acts in the nucleus. Transgenic *Arabidopsis *overexpressing *GhMPK16 *displayed significant resistance to fungi (*Colletotrichum nicotianae *and *Alternaria alternata*) and bacteria (*Pseudomonas solanacearum*) pathogen, and the transcripts of pathogen-related (PR) genes were more rapidly and strongly induced in the transgenic plants. Furthermore, transgenic *Arabidopsis *showed reduced drought tolerance and rapid H_2_O_2 _accumulation.

**Conclusion:**

These results suggest that *GhMPK16 *might be involved in multiple signal transduction pathways, including biotic and abiotic stress signaling pathways.

## Background

Stresses such as drought, high salinity and fungal infections constitute a major limitation to crop productivity. Plants have developed sophisticated defense mechanisms to deal with diverse unfavorable environmental factors. The mitogen-activated protein kinase (MAPK) cascades are conserved pathways through which extracellular stimuli are transduced into intracellular responses in all eukaryotes [1,2]. Plant MAPK cascades have been shown to regulate a number of essential biological processes, including growth, development and stress responses [3].

MAPK cascades are composed of three interlinked protein kinases: MAPKK kinases (MAPKKKs or MAP3Ks), MAPK kinases (MAPKKs, MAP2Ks or MEKs) and MAPKs. MAPKs are the terminal components in this cascade, and they are regulated by the dual phosphorylation of the conserved T-X-Y motif located in the activation loop by upstream kinases (MAPKKs). There are 20 MAPK genes identified in *Arabidopsis*, and a similar repertoire of genes have been found in other plants, such as rice (*Oryza sativa*), poplar (*Populus *sp.) and grapevine (*Vitis vinifera*) [3-5]. The MAPKs can be categorised into four major groups (A, B, C, and D) based on the phylogenetic analyzes of amino acid sequences and phosphorylation motifs (TEY and TDY). The TEY subtype can be classified into three groups (A, B and C), whereas the TDY subtype is found in the more distant group D [4,5].

In *Arabidopsis*, three particular MAPKs in groups A and B (MPK3, MPK4 and MPK6) have been extensively studied. Both biochemical and genetic analyzes have been performed for each of these isoforms, which appear to work in multiple signaling pathways and play crucial roles in many distinct processes ranging from stress responses to developmental processes [3]. Information about group C MAPKs has recently emerged. Three members of group C, *MPK1 *and *MPK2 *in *Arabidopsis *and *PsMPK2 *in pea (*Pisum sativum *L.), are transcriptionally induced by a variety of stresses [6,7]. More recently, *Arabidopsis *group C MAPKs, including MPK1, MPK2, MPK7 and MPK14, were reported to be activated by MKK3, and MKK3-MPK7 participates in pathogen signaling [8]. Cotton *GhMPK7 *may play a role in pathogen resistance, plant growth and development [9].

Based on the phylogenetic analysis and pairwise comparison of *Arabidopsis *and rice MAPKs, it has been proposed that the rice genome contains more MAPKs with a TDY phosphorylation motif (11 members) than with a TEY motif (6 members). In contrast, the *Arabidopsis *genome contains more MAPKs with a TEY motif (12 members) than with a TDY motif (8 members) [10]. Detailed functional data about TDY MAPKs was first obtained from a monocot plant. Overexpression of *OsBWMK1 *(also known as *OsMPK12*) in tobacco resulted in constitutive PR gene expression and enhanced resistance to fungal and bacterial infections [11]. In maize, ZmMPK6 is able to interact with a 14-3-3 protein, and these data represent the first evidence of the possible involvement of 14-3-3 proteins in the regulation of MAPK cascades in plants [12]. More recently, *Arabidopsis MPK9 *(a group D MAPK) and *MPK12 *(a group B MAPK) were found to be preferentially expressed in guard cells, share functional redundancy and function as positive regulators downstream of reactive oxygen species (ROS) in guard cell abscisic acid (ABA) signaling [13]. Moreover, *Arabidopsis *MPK18 helps to mediate cortical microtubule functions in plant cells [14].

Cotton (*Gossypium hirsutum*) is one of the most important fibre and oil crops, and its growth and yield are severely impaired in various biotic/abiotic stress conditions. The biological significance of cotton group D MAPKs has not yet been described. In this study, a cDNA clone, *GhMPK16*, encoding a putative group D MAPK gene was isolated and characterised. Our results indicate that the expression of *GhMPK16 *is induced by chemical and biological signals. Ectopic expression of *GhMPK16 *in *Arabidopsis *results in enhanced disease resistance against fungi and bacteria pathogen. Moreover, *GhMPK16 *transgenic plants were obviously more drought-sensitive than control plants. We deduced that *GhMPK16 *may play important roles in regulating pathogen resistance and drought signaling.

## Results

### Cloning and characterisation of the full-length *GhMPK16 *cDNA

Based on the conserved region of plant group D MAPK genes, the degenerate primers MP1 and MP2 were designed and synthesized to clone the internal conserved region of MAPKs from cotton, and a putative MAPK fragment (476 bp) was cloned. Next, rapid amplification of cDNA ends-PCR (RACE-PCR) was performed, and the full length sequence was retrieved. A 3'-end fragment of about 1500 bp and a 5'-end fragment of about 360 bp were obtained with the specific primers. The full-length cDNA sequence was PCR amplified using the gene-specific primers (FL1 and FL2), and it showed a high degree of homology to group D MAPKs especially *Arabidopsis AtMPK16*. According to the nomenclature for plant MAPKs, the novel cotton *MAPK *gene was named *GhMPK16 *[4,5], and is the first group D MAPK identified in cotton. The full-length cDNA sequence of *GhMPK16 *(GenBank accession number: FJ966889) was 2030 bp with a 78 bp 5' untranslated region (UTR) and a 287 bp 3' UTR. The cDNA contains a 1665 bp open reading frame (ORF) that is predicted to encode a protein of 554 amino acids with a predicted molecular mass of 63.18 kDa and an isoelectric point (pI) of 8.61.

**Figure 1:**
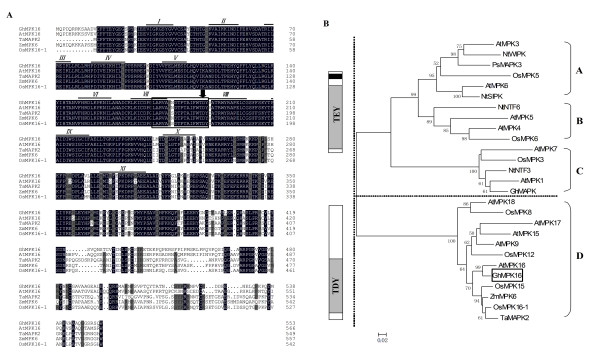
**Sequence analysis of GhMPK16**. (A) Alignment of the deduced GhMPK16 protein sequence with other known plant group D MAPKs. Amino acid sequences used in the analysis are from *Arabidopsis thaliana *(AtMPK16, NP_197402), *Triticum aestivum *(TaMAPK2, ABC54585), *Zea mays *(ZmMPK6, NP_001105238), *Oryza sativa *(OsMPK16-1, ACF06191), *Gossypium hirsutum *(GhMPK16, FJ966888). These sequences were aligned using the DNAman 6.0 program. Identical and similar amino acids were shaded in black and gray, respectively. The protein kinase subdomains are indicated by Roman numerals (I to XI) over the sequences. The activation-loop is boxed, and the phosphorylation motif (TDY) is marked by an arrow. (B) The phylogenetic relationships of GhMPK16 and other plant MAPKs. Numbers above or below the branches indicate bootstrap values (>50%) from 1,000 replicates. The amino acid sequences of MAPKs used for construction of the tree are deposited to the GenBank database under the following accession numbers: AtMPK3 (NP_190150); NtWIPK (BAA09600); PsMAPK3 (AAF73236); OsMPK5 (AAL87689); AtMPK6 (NP_181907); NtSIPK (AAB58396); NtNTF6 (CAA58760); AtMPK5 (NP_567378); AtMPK4 (NP_192046); OsMPK6 (NP_922504); AtMPK7 (NP_179409); OsMPK3 (ABH01189); NtNTF3 (CAA49592); AtMPK1 (NP_172492); GhMAPK (ABA00652); AtMPK18 (NP_175756); OsMPK8 (CAD54742); AtMPK17 (NP_001030941); AtMPK15 (NP_565070); AtMPK9 (NP_566595); OsMPK12 (AAD52659); AtMPK16 (NP_197402); GhMPK16 (FJ966889); OsMPK15 (ACD76441); ZmMPK6 (NP_001105238); OsMPK16-1 (ACF06191); TaMAPK2 (ABC54585). At, *Arabidopsis thaliana*; Nt, *Nicotiana tobacum*; Ps, *Pisum sativum*; Ta, *Triticum aestivum*; Zm, *Zea mays*; Os, *Oryza sativa*; Gh, *Gossypium hirsutum*. The Ser/Thr kinase domain is indicated as gray and contains part of the activation-loop motif; the CD domain required for MAPK docking is indicated in black. GhMPK16 is boxed in the diagram and classified as a member of the TDY group.

A BLAST search (http://www.ncbi.nlm.nih.gov/BLAST) and multi-alignment analysis revealed that GhMPK16 is highly related to other group D MAPKs, sharing a homology of 81.48% to AtMPK16, 72.18% to TaMAPK2, 74.56% to OsMPK16-1 and 76.02% to ZmMPK6 (Figure 1A). Moreover, as with other known plant group D MAPKs, GhMPK16 has the same family signature of 11 conserved subdomains, an activation loop (T-loop), a phosphorylation motif (TDY motif) in the T-loop, and an extended C-terminal region relative to groups A, B and C. Additionally, GhMPK16 also lacks a C-terminal CD domain, a feature conserved in MAPKs belonging to the other groups (A, B, C) [4].

To reveal the evolutionary relationship of GhMPK16 with other MAPKs from various plant species, we constructed a phylogenetic tree using amino acid sequences derived from the GenBank database (Figure 1B). Our results indicate that GhMPK16 has high similarity with AtMPK16 and a close genetic relationship with many monocot (rice, corn, and wheat) group D MAPKs, such as OsMPK16-1, OsMPK15, ZmMPK6 and TaMAPK2, which suggests that these genes may have a similar function across species including dicotyledons and monocotyledons.

### Genomic structure and Southern blot analysis of *GhMPK16*

To isolate a genomic *GhMPK16 *clone, two pairs of specific primers, FL1/Z1 and FL2/Z2, were designed based on the *GhMPK16 *cDNA sequence, and the cotton genomic DNA was used as the template to generate two PCR fragments. These two fragments were further linked together through their overlapping region and the full-length *GhMPK16 *genomic sequence of 5520 bp (GenBank accession number: FJ966896) was deduced. A comparison between the *GhMPK16 *genomic and cDNA sequences indicated that ten introns were present in the gene (Additional file 1: supplementary Figure S1). Interestingly, exon 8 was only 5 bp, intron 7 did not contain a 3'-splice acceptor AG signal, and intron 8 did not contain the conserved 5'-splice donor GT signal. A comparative analysis of the homologous genes from *Arabidopsis thaliana*, *Oryza sativa *and *Vitis vinifera *was performed (Additional file 1: supplementary Figure S1). This analysis indicated that the size of the exons was conserved, but the length of the introns for each member was different. To date, the most noticeable difference in *GhMPK16 *was the number of exons. *GhMPK16 *has 11 exons, and the others have 10.

**Figure 2:**
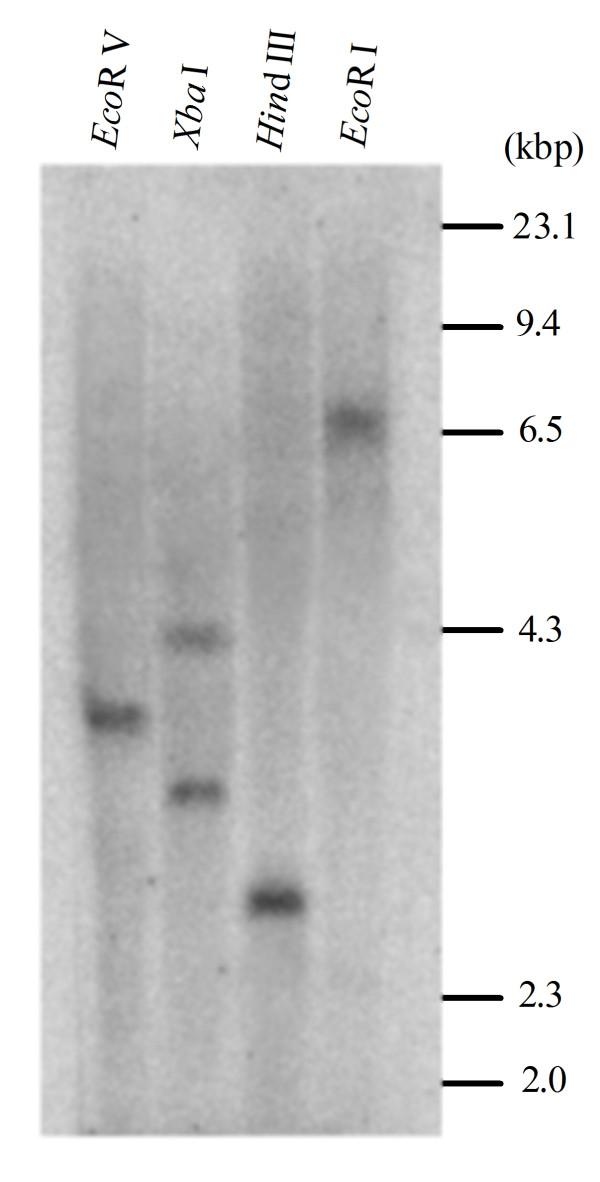
**Southern blot analysis for *GhMPK16 *in the cotton genome**. Genomic DNA (30 mg/sample) was digested with *Eco*R V, *Xba *I, *Hin*d III and *Eco*R I respectively, followed by hybridization with the partial α-^32^P -labeled genomic *GhMPK16 *fragment. Molecular weight marker is shown on the right.

Southern blots were used to investigate the genomic organization of the *GhMPK16 *gene. Genomic DNA was completely digested with *Eco*R V, *Xba *I, *Hin*d III and *Eco*R I and hybridised to the 3' partial sequence of *GhMPK16*, which contains no restriction sites of *Eco*R V, *Hin*d III and *Eco*R I and only one *Xba *I site presents in the probe region. As shown in Figure 2, only one band was observed for the *Eco*R V, *Hin*d III and *Eco*R I digestions, and two bands were observed for the *Xba *I digestion. These results imply that there is a single *GhMPK16 *gene in the cotton genome.

### Subcellular localization of GhMPK16

**Figure 3:**
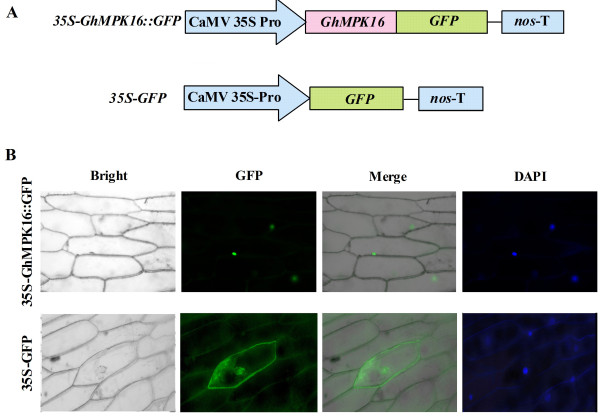
**Subcellular localization of the GhMPK16 protein in onion epidermal cells**. (A) Schematic diagram of the *35S-GhMPK16::GFP *and *35S-GFP *fusion construct. (B) Transient expression of *35S-GFP *and *35S-GhMPK16::GFP *in onion epidermal cells. Cells were analyzed by laser confocal microscopy 12 h after particle bombardment. The nuclei of the onion cells were visualised by DAPI staining. Bar = 20 μm.

To reveal the cellular localization of GhMPK16, a reporter gene encoding GFP was fused to *GhMPK16 *and placed under the control of the CaMV35S promoter, and immunoblot analysis indicated that GhMPK16::GFP was an integrated fusion protein (Additional file 2: supplementary Figure S2). The biolistic transformation system was used for a transient assay in onion epidermal cells. The nuclear localization of GFP-conjugated GhMPK16 was confirmed in individual transgenic cells by GFP fluorescence, using DAPI staining to detect the nuclei and interference contrast images to detect whole-cell structures. As shown in Figure 3B, the *35S-GhMPK16::GFP *construct localized to the nucleus, and the 35S-GFP control construct showed GFP signals in both the cytoplasm and the nucleus. In addition, a program that predicts the subcellular localization of proteins (http://www.bioinfo.tsinghua.edu.cn/SubLoc/) predicted that GhMPK16 is localized in the nucleus with an expected accuracy of nearly 74%. These results indicate that the GhMPK16 protein is likely localized in the nucleus.

### Expression pattern of *GhMPK16 *under diverse abiotic and biotic stresses in cotton

**Figure 4:**
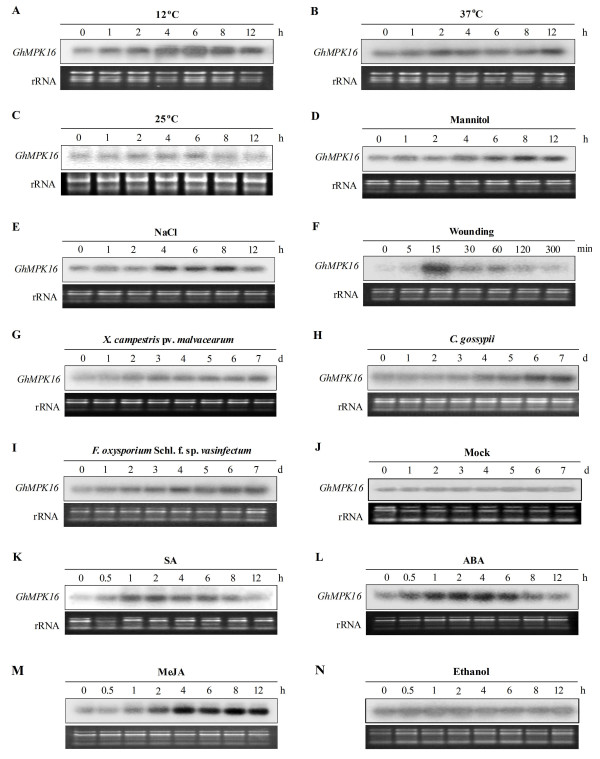
**Expression profiles of *GhMPK16 *under abiotic/biotic stresses**. RNA blots were performed with total RNA extracted from leaves at the indicated times, and treated with low temperature (12°C) (A), high temperature (37°C) (B), normal temperature (25°C) (C), 200 mM mannitol (D), 200 mM NaCl (E), wounding (F), *X. campestris *pv. *Malvacearum *(G), *C. gossypii *(H), *F*. *oxysporum *f. sp. *Vasinfectum *(I), mock (J), 10 mM SA (K), 100 μM ABA (L), 100 μM MeJA (M), or ethanol (N). Ethidium bromide-stained rRNA was used as the loading control.

To determine whether *GhMPK16 *expression is triggered by abiotic stresses, cotton seedlings were exposed to low and high temperatures, mannitol, NaCl and wounding treatments. *GhMPK16 *showed a slightly response to low and high temperatures (Figure 4A, B, C). *GhMPK16 *transcripts accumulated within 6 h following mannitol treatment, and the induction was still present 12 h after treatment (Figure 4D). As shown in Figure 4E, *GhMPK16 *expression increased and reached the maximum at 8 h after the NaCl treatment. Wounding rapidly and transiently enhanced the expression level of *GhMPK16 *(Figure 4F). Taken together, the expression profiles indicate that *GhMPK16 *is induced by various abiotic stresses.

To explore the roles of *GhMPK16 *in plant biotic stresses, cotton seedlings were inoculated with pathogens, including *Xanthomonas campestris *pv. *malvacearum *(*X. campestris *pv. *malvacearum*), *Colletotrichum gossypii *(*C. gossypii*) and *Fusarium oxysporum *f. sp. *vasinfectum *(*F. oxysporum *f. sp. *vasinfectum*). As shown in Figure 4 (G, H, I), infection by all of these pathogens elevated the transcription level of *GhMPK16*, although the induction kinetic was variable. There was no significant change in *GhMPK16 *transcript level without pathogen treatment during 7 days (Figure 4J). These results indicate that *GhMPK16 *may be intimately involved in the plant pathogen defense response.

Additionally, we examined the response of *GhMPK16 *to exogenously applied salicylic acid (SA), methyl jasmonate (MeJA) and ABA, which are plant signaling molecules involved in plant defense signaling pathways. The expression of *GhMPK16 *was induced by all of these signal molecules (Figure 4K, L, M). Furthermore, the solvent control (Ethanol) did not significantly induce the expression of *GhMPK16 *(Figure 4N)

### *GhMPK16 *promoter analysis

**Figure 5:**
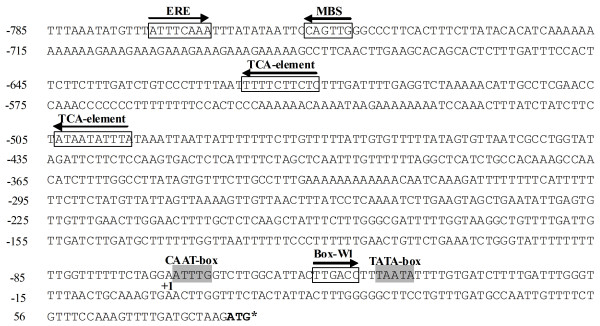
**Nucleotide sequence of the promoter region of *GhMPK16***. The predicted transcription initiation site is indicated (+1, A). The start codon is marked with an asterisk, and the putative core promoter consensus sequences (TATA-box and CAAT-box) are highlighted in grey. The putative *cis*-acting elements are indicated by boxes and their corresponding names are given above each element. Arrows indicate the direction of the *cis*-element. Box-W1 is a fungal elicitor responsive element, ERE is an ethylene-responsive element, the MBS binding site is involved in gene induction in response to drought-inducibility and the TCA-element is a *cis*-acting element involved in SA-responsive.

Inverse-PCR (I-PCR) was used to obtain a 785 bp fragment of the 5' flanking region upstream of the transcriptional start site, as determined by the *GhMPK16 *cDNA sequence. In order to find *cis*-acting elements, the PlantCARE databases were analyzed. Sequence analysis revealed that the *GhMPK16 *promoter contains TATA and CAAT motifs located at nucleotides -38 and -64 relative to the transcriptional start site, respectively, which is characteristic of eukaryotic gene promoters. As shown in Figure 5, the 785 bp promoter region contains several motifs probably related to pathogen and drought signals, such as a Box-W1 element (fungal elicitor responsive element), an ERE element (ethylene-responsive element), two TCA-element (*cis*-acting SA-responsive element) and a MBS element (MYB binding site involved in drought-inducibility). This suggests that these putative *cis*-acting elements are responsible for enhanced expression of *GhMPK16 *during stress conditions.

### Enhanced resistance of *GhMPK16 *transgenic plants to pathogenic infections

**Figure 6:**
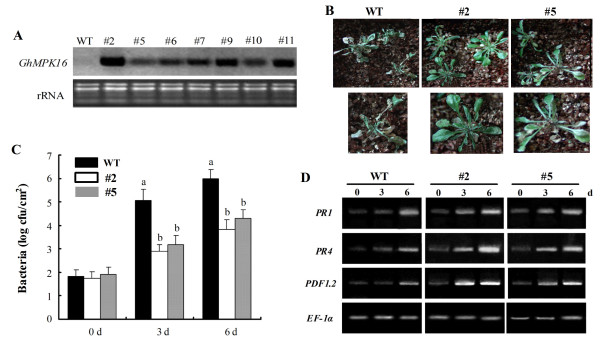
**Enhanced resistance of *GhMPK16*-overexpressing *Arabidopsis *to *P. solanacearum *infection**. (A) RNA blot analysis of *GhMPK16 *expression in T_1 _transgenic plants. (B) Disease symptoms of wild-type (WT) and transgenic plants (#2 and #5) at 6 d post-inoculation with *P. solanacearum *(10^5 ^cfu/ml). Magnified images are shown in the lower photographs. (C) Growth of *P. solanacearum *in wild-type and transgenic plants (#2 and #5) after infecting the plants using the dipping method. Data are the mean ± SE (n = 6) from three independent experiments. Different letters at 3- or 6-d data points indicate significant differences with P < 0.05 according to Duncan's multiple range test. (D) RT-PCR analysis of the expression of *Arabidopsis AtPR1*, *AtPR4 *and *AtPDF1.2 *genes in wild-type and transgenic plants (#2 and #5) inoculated with *P. solanacearum*. *Arabidopsis *elongation factor *EF-1α *was used as an internal control.

In order to further explore the function of *GhMPK16 *in plant defense, the coding sequence of *GhMPK16 *was cloned into the plant binary vector pBI121 and transformed into *Arabidopsis*. A total of 32 independent transgenic lines were obtained by kanamycin-resistance selection and confirmed by PCR (data not shown). RNA blot analysis was performed to determine the transgenic expression levels in 7 randomly selected lines (Figure 6A). Two representative lines (#2 and #5) exhibiting different expression levels were selected and their T_3 _transgenic plants used to evaluate disease resistance.

To analyze bacterial resistance responses in wild-type and *GhMPK16*-overexpressing plants, 3-week-old plants were inoculated with *P. solanacearum *and monitored daily for the appearance of typical disease symptoms. *P. solanacearum *caused more severe chlorotic symptoms in the wild-type plants compared to the transgenic plants 6 d after inoculation (Figure 6B). As shown in Figure 6C, the bacterial titres in the inoculated leaves of the transgenic plants were significantly reduced as compared to those in wild-type plants at 3- and 6-day post-inoculation, which is consistent with the observed symptoms. The rate of bacterial growth in the inoculated leaves was lower for transgenic line #2 than for transgenic line #5. These results indicate that *GhMPK16 *can enhance resistance to *P. solanacearum *in a dose-dependent manner in transgenic plants. In order to better understand the role of *GhMPK16 *in disease resistance in transgenic plants, three marker genes associated with pathogen responses (*AtPR1*, *AtPR4*, and *AtPDF1.2*) were selected, and the transcript levels of those genes were analyzed by reverse transcription-PCR (RT-PCR). The expression of the three marker genes, especially *AtPR4 *and *AtPDF1.2*, was more rapidly and strongly induced by the *P. solanacearum *infection in transgenic plants than in wild-type plants (Figure 6D). These results demonstrate that the overexpression of *GhMPK16 *activates defense-related gene expression when plants are challenged with *P. solanacearum*.

**Figure 7:**
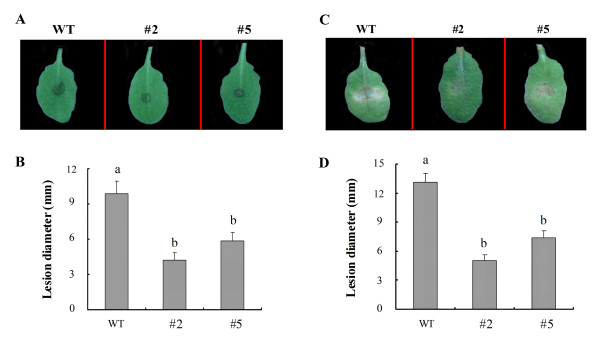
**Inhibition of fungal pathogen infections in *GhMPK16*-overexpressing *Arabidopsis *lines**. (A) Disease symptoms of leaves from wild-type (WT) and transgenic plants (#2 and #5) 5 d after inoculation with *C. nicotianae *(10^6 ^conidia/ml). (B) The diameter (mm) of the lesions was measured 5 d after inoculation with *C. nicotianae*. Data are the mean ± SE (n = 6) from three independent experiments. Different letters indicate significant differences with P < 0.05 according to Duncan's multiple range test. (C) Disease symptoms of the leaves from wild-type (WT) and transgenic plants (#2 and #5) 5 d after inoculation with *A. alternate *(10^6 ^conidia/ml). (D) The diameter (mm) of the lesions was measured 5 d after inoculation with *A. alternate*. Data are the mean ± SE (n = 6) from three independent experiments. Different letters indicate significant differences with a P < 0.05 according to Duncan's multiple range test.

As shown in Figure 7, *GhMPK16*-overexpressing plants were evaluated for their resistance to fungal pathogens (*C. nicotianae *and *A. alternate*). Detached leaves were inoculation with *C. nicotianae *and *A. alternate*, and after 5 days, the lesions in the wild-type leaves were larger than those in the transgenic leaves, and line #2, which displayed the highest level of *GhMPK16 *expression, exhibited the least severe disease symptoms. To quantify the lesions, the diameters of the disease spots were measured, and the data were consistent with the visual observations. These results indicate that *GhMPK16 *can enhance resistance to infections by pathogenic fungi in transgenic plants. After inoculating whole plants with *C. nicotianae *and *A. alternate*, the expression of *AtPR1*, *AtPR4 *and *AtPDF1.2 *was observed at 3- and 6-day post-infection in both the wild-type and transgenic plants, and the expression patterns of the three marker genes were similar to those observed with the *P. solanacearum *challenge (data not shown).

### Reduced drought tolerance and rapid H_2_O_2 _accumulation in *GhMPK16*-overexpressing plants

**Figure 8:**
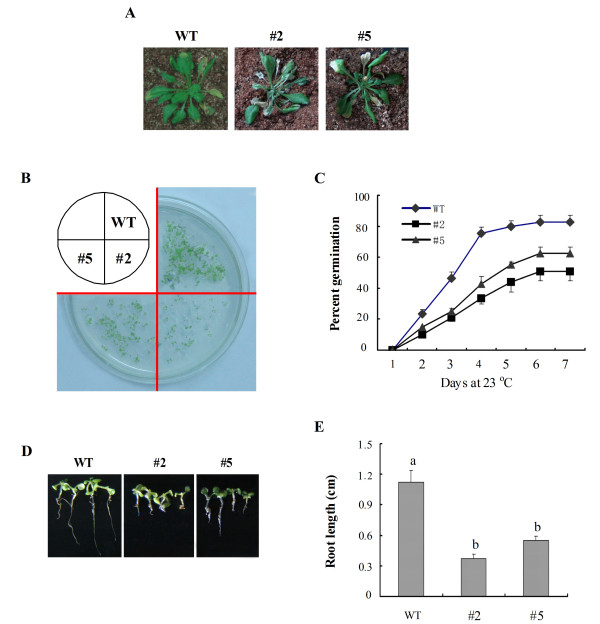
**Drought stress analysis of *GhMPK16*-overexpressing *Arabidopsis***. (A) Phenotypes of wild-type (WT) and transgenic plants (#2 and #5) plants withheld from water for 15 d at the vegetative stage. (B) Seed germination of wild-type (WT) and transgenic plants (#2 and #5) after 4 days on 1/2 MS agar medium containing 50 mM mannitol. (C) Germination percentage of seeds on 1/2 MS agar medium containing 50 mM mannitol. Germination was defined as the emergence of the root radical and was scored daily. Data are the mean ± SE (n = 4) from three independent experiments. (D) Growth of wild-type (WT) and transgenic plant (#2 and #5) seedlings after 100 mM mannitol treatment. Photos were taken 10 d after mannitol treatment. (E) Root length of wild-type (WT) and transgenic plant (#2 and #5) seedlings after 100 mM mannitol treatment for 12 d. Data are the mean ± SE (n = 6) from three independent experiments. Different letters indicate significantly different at P < 0.05 according to Duncan's multiple range test.

Compared with wild-type plants, the *GhMPK16*-overexpressing plants exhibited obvious drought sensitivity. As shown in Figure 8A, the leaves began to turn yellow and curl in both the 3-week-old wild-type and transgenic plants after a 15-day drought treatment. Additionally, drought treatment led to more serious damage in the transgenic plants than in the wild-type plants. Under normal conditions, no overt morphological differences were observed between wild-type and transgenic plants (data not shown). We tested the seed germination capacity on 1/2 MS agar medium with 50 mM mannitol to mimic drought conditions and found that the germination percentage was higher for wild-type plants than for transgenic plants (Figure 8B, C). The root lengths of both wild-type and transgenic plants were used as indicators of drought stress tolerance. Root growth was inhibited by mannitol treatment to a greater extent in transgenic seedlings than in wild-type seedlings (Figure 8D, E). These results suggest that the overexpression of *GhMPK16 *in *Arabidopsis *results in reduced drought tolerance.

**Figure 9:**
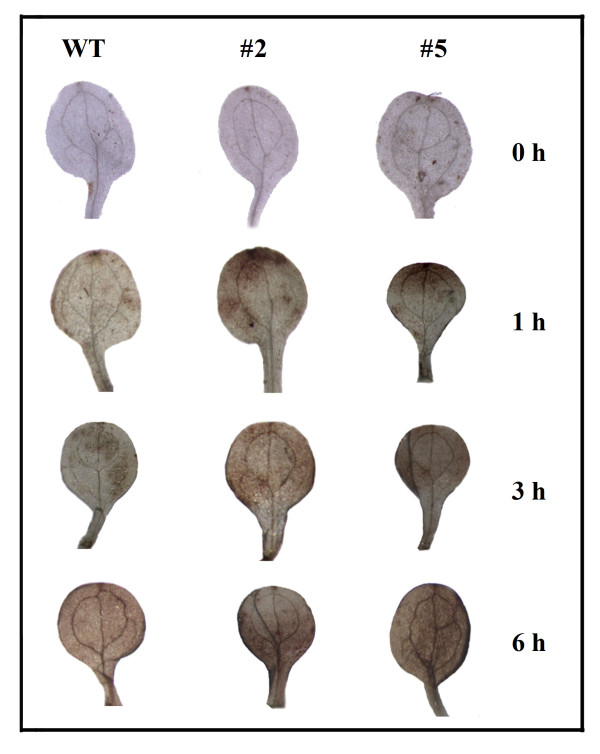
**DAB coloration assay of H**_**2**_**O**_**2 **_**accumulation in wild-type (WT) and*GhMPK16*-overexpressing transgeni**c **plants after 200 mM mannitol treatment**. Mannitol was used to treat one-week-old plants for 0, 1, 3 or 6 h.

One of the important responses of plants under osmotic stress is the accumulation of reactive oxygen species (ROS). Among the different ROS, only H_2_O_2 _can cross plant membranes and thus directly function in cell-to-cell signaling. Therefore, we examined endogenous H_2_O_2 _accumulation in wild-type and transgenic plants in response to mannitol treatment. Mannitol (200 mM) was used to treat one-week-old seedlings, and the leaves were collected after 0, 1, 3 and 6 h. Staining with 3,3'-diaminobenzidine (DAB) revealed different levels of H_2_O_2 _production in the leaves of wild-type and transgenic plants after osmotic stress treatment. Specifically, H_2_O_2 _accumulated in transgenic plants at a significantly faster speed than that in wild-type plants (Figure 9). These results suggest that the overexpression of *GhMPK16 *leads to the rapid production of ROS or ineffective scavenge of excess ROS after osmotic stress.

## Discussion and Conclusion

In this study, we isolated and characterised *GhMPK16*, a gene whose product belongs to the family of group D MAPKs. *GhMPK16 *is the first characterized MAPK gene from cotton belonging to the group D MAPKs. The corresponding protein is characterized by the presence of a TDY activation motif in its T-loop, the lack of a CD domain and an extended C-terminal region compared to the TEY subtypes of MAPKs (Figure 1A). These structural differences suggest that group D MAPKs may function differently from other MAPKs.

The subcellular localization of group D MAPKs has not been established. OsBWMK1, a group D MAPK from rice, is found in the nucleus and mediates pathogenesis-related gene expression by activating the OsEREBP1 transcription factor [11]. Intriguingly, the alternative splicing of *OsBWMK1 *generates three different transcriptional variants that produce proteins with different subcellular localizations [15]. In *Arabidopsis*, AtMPK18 is located in the cytoplasm [14]. Analysis of the subcellular localization of a GFP-tagged GhMPK16 protein in transiently transformed onion epidermal cells revealed that GhMPK16 is likely localized in the nucleus (Figure 3). Therefore, we speculated that GhMPK16 may act as a transcriptional activator by activating the expression of a set of target genes in the nucleus.

Gene expression patterns are usually an indicator of gene function. Notably, *GhMPK16 *can be induced by various pathogens, including *X. campestris *pv. *malvacearum*, *R.solani *and *C. gossypii*. RNA blot analyzes revealed that the expression of *GhMPK16 *could be induced by signal molecules such as SA, MeJA, and ABA (Figure 4). These results imply that *GhMPK16 *may play a role in plant defense responses and in the regulation of certain components of multiple stress-signaling pathways. Consistent with this hypothesis, sequence analysis of the *GhMPK16 *promoter revealed several motifs, such as Box-W1 element, ERE element and TCA-element, related to motifs with important roles in defense signaling.

Recently, a variety of MAPK genes have been identified and subsequently explored by both genetic and biochemical approaches. Increasing evidence has shown that the MAPK cascade is an important pathway in pathogen-triggered signal transduction [16,17]. Signal molecules such as SA, jasmonic acid (JA) and ethylene (ET), regulate distinct sets of pathogenesis-related (PR) genes in different pathogen defense pathways. SA and MeJA are involved in two major defense signaling pathways, the SA-dependent and JA/ET-dependent defense mechanisms, which act against different types of pathogens [18]. In *Arabidopsis*, *AtMPK4 *responds to the balance between SA and MeJA through the EDS1/PAD4 module and regulates the SA and JA/ET-related defense responses [19]. When GhMPK16 is ectopically expressed in *Arabidopsis*, the transgenic *Arabidopsis *show enhanced disease resistance against bacteria (*P. solanacearum*) and fungi (*C. nicotianae *and *A. alternate*) (Figure 6, 7). *PR1 *is known as a marker gene for the SA signaling pathway, and *AtPR4 *and *AtPDF1.2 *are markers for the JA/ET signaling pathway. Along with the enhanced disease resistance in *GhMPK16*-overexpressing plants, the expression of the examined defense-related genes *AtPR1*, *AtPR4*, and *AtPDF1.2 *was elevated. Thus, it is reasonable to speculate that *GhMPK16 *may be involved in the crosstalk between the SA- and JA/ET- mediated pathogen defense pathways.

*GhMPK16 *showed a response to various abiotic stresses, such as low or high temperatures, mannitol and NaCl (Figure 4). Interestingly, transgenic plants overexpressing *GhMPK16 *exhibited obvious drought sensitivity (Figure 8). *GhMPK16 *overexpression resulted in a significant reduction in both germination percentage and root growth after mannitol treatment, and seedling growth was more severely inhibited by drought stress in *GhMPK16*-overexpressing plants than in wild-type plants. ABA responds to the environmental signals to protect plants from abiotic stresses, such as cold, drought and salt stresses [20,21]. The expression of *GhMPK16 *was strongly induced by exogenous ABA and the *GhMPK16*-overexpressing plants were sensitive to drought tolerance comparing with wild-type *Arabidopsis*, suggesting that *GhMPK16 *may be involved in ABA signaling and caused negative function for drought tolerance. DAB staining revealed that H_2_O_2 _rapidly accumulation in *GhMPK16*-overexpressing transgenic plants after drought treatment (Figure 9). Excessive ROS can injure plants. *GhMPK16 *accelerates the accumulation of H_2_O_2_, and this may be the reason that drought causes more serious damage in the transgenic plants. In *Arabidopsis*, *MPK9 *and *MPK12 *function downstream of ROS to regulate guard cell ABA signaling positively [13]. Guard cell-specific inhibition of *Arabidopsis MPK3 *expression causes abnormal stomatal responses to ABA and H_2_O_2 _[22]_. _More physiological, biochemical and molecular experiments are needed to elucidate the mechanism of *GhMPK16 *in response to drought stress.

On the basis of these observations, we propose that GhMPK16 functions in at least two signaling pathways, one that responds to pathogens and another that is involved in drought stress. *GhMPK16 *may serve as a point for crosstalk between biotic and abiotic stress response signaling. Further studies on the function and regulation of GhMPK16 are required.

## Methods

### Plant materials and treatments

The cotton cultivar *Gossypium hirsutum *L. cv Lumian 22 was used for all of the experiments. Germinated seedlings were grown by aquaculture in tissue culture pots (sterile water) under greenhouse conditions at 25°C with a 16 h light/8 h dark cycle. The following treatments were performed on seven-day-old cotton seedlings: 10 mM Salicylic acid (SA), 100 μM methyl jasmonate (MeJA) (ethanol as a solvent control) or 100 μM abscisic acid (ABA) was sprayed onto leaves of cotton seedlings; seedling roots were placed in 200 mM NaCl and 200 mM mannitol solutions for salt and drought stresses, respectively; seedling leaves were cut with scissors for wound treatment; seedlings were placed in a growth chamber at 37°C or 12°C for the high and low temperature treatments, respectively; roots were dipped into suspensions of the bacterial pathogen *Xanthomonas campestris *pv. *malvacearum *(*X. campestris *pv. *malvacearum*) (OD = 0.001) to cause a bacterial infection and the fungal pathogens *Fusarium oxysporum *f. sp. *vasinfectum *(*F. oxysporum *f. sp. *vasinfectum*) and *Colletotrichum gossypii *(*C. gossypii*) conidial suspensions (10^5 ^conidia/ml) in 1% glucose were dropped onto plant leaves to cause the fungal infections. The plants treated with 1% glucose were considered as mock. The leaves, roots and stems were harvested at the appropriate time points as indicated and frozen in liquid nitrogen for further analyzes.

*Arabidopsis *(ecotype Columbia, Col-0) and transgenic *Arabidopsis *seeds were planted directly in soil or transplanted after germinating on 1/2 MS agar medium and grown in a chamber under SD (8 h light/16 h dark) or LD (16 h light/8 h dark) conditions at 23°C. All seeds were treated at 4°C for 2 d before being transferred to a growth chamber. *Arabidopsis *were grown in SD conditions for 2-week and then transferred to LD conditions. 3-week-old *Arabidopsis *plants were used for the disease resistance assays. The bacterial pathogen *Pseudomonas solanacearum *(*P. solanacearum*) was cultivated in King's B medium at 30°C for 2 days, and the bacterial cells were resuspended in 10 mM MgCl_2 _to a concentration of 10^5 ^colony-forming units/ml (cfu/ml). Plants were infected using the dipping method, and bacterial titres were assessed by plating a dilution series of leaves ground in 10 mM MgCl_2 _on King's B medium as described by Liu et al. [23]. Infected leaves were sampled immediately and at 3- and 6-d after inoculation. To evaluate resistance against the fungal pathogens *Colletotrichum nicotianae *(*C. nicotianae*) and *Alternaria alternate *(*A. alternate*), two methods were used: the detached leaf inoculation assay and the whole plant inoculation assay. A spore suspension (10^6 ^conidia/ml) was prepared in 1% glucose. In the detached leaf inoculation assay, leaves were inoculated with a 10 μl droplet of conidial suspension. In the whole plant inoculation assay, a vaporiser was used to inoculate the plants with the conidial suspension. The inoculated leaves and plants were kept in a moist chamber under dark conditions at 25°C for 48 h and then incubated under LD conditions at 25°C.

For the drought stress tolerance analysis, *Arabidopsis *were grown in SD conditions for 2-week and then transferred to LD conditions. For 15 d, 3-week-old *Arabidopsis *plants were completely withheld from water. Additionally, *Arabidopsis *seeds were germinated in 1/2 MS agar medium or 1/2 MS agar medium supplemented with 50 mM mannitol. Analysis of the growth of transgenic *Arabidopsis *after mannitol treatment, *Arabidopsis *seeds germinated in 1/2 MS agar medium and incubated for 3 d. The seedlings were then carefully transferred into 1/2 MS agar medium supplemented with 100 mM mannitol, and root lengths were measured over the next 10 d.

### Cloning of the *GhMPK16 *gene

Total RNA was extracted from cotton seedling leaves using TRIzol Reagent (Invitrogen, Carlsbad, CA, USA) according to the manufacturer's protocol. The full-length *GhMPK16 *cDNA was amplified by reverse transcription-PCR (RT-PCR) and rapid amplification of cDNA ends-PCR (RACE-PCR).

The first cDNA strand was synthesised using approximately 2 μg of total RNA, the Oligo(dT)_18 _adaptor primer and M-MLV reverse transcriptase (Promega, Madison, WI, USA) for 1 h at 42°C. To clone the internal conserved fragment, primers MP1 and MP2 were designed and synthesised based on the conserved amino acid and nucleotide sequences of the plant group D MAPK genes. Cloning was performed as described previously [24], and the primer sequences are provided in Table 1.

**Table 1:** The primers used in this study

**Abbreviation**	**Sequence (5'--3')**	**Description**
MP1	AARGGNAGYTAYGGNGTNGT	Degenerate primer, forward
MP2	GCNACRTARTCNGTCCARAA(H = A, C, or T; N = A, C, G, or T; R = A or G; V = A, C, or G; Y = C or T)	Degenerate primer, reverse
3P1	TTCTTTATCAGCTTCTTCGGG	3' RACE reverse primer, outer
3P2	TTCATCGGGATCTAAAGCCG	3' RACE reverse primer, nested
B26	GACTCGAGTCGACATCGAT (T)_18_	Universal primer, outer
B25	GACTCGAGTCGACATCGAT	Universal primer, nested
5P1	GAAGGAGGGAACAATATGTGC	5' RACE reverse primer, outer
5P2	ATGACGTAGGAGCCTGAGAAG	5' RACE reverse primer, nested
AAP	GGCCACGCGTCGACTAGTAC (G)_14_	Abridged anchor primer
AUAP	GGCCACGCGTCGACTAGTAC	Abridged universal amplification primer
FL1	GATGCTAAGATGCAGCCTGATC	Full-length cDNA primer
FL2	GCTTCACTTGTAACTTGTCTGAGC	Full-length cDNA primer
Z1	CCGCACTTGGATACATGAAGCC	Genomic sequence primer
Z2	GAGTATCTGGAAGGATCAGAGCC	Genomic sequence primer
5P3	GCTGCCAGACCTGGGAAAGTAG	primer used for probe synthesized
Wup1	CACGCCATTAAACCAAGTAG	I-PCR primer
Wdra1	GAGGTTGACTAACACTAGTG	I-PCR primer
Wup2	GATCAGGCTGCATCTTAGCATC	I-PCR primer
Wdra2	AGCGTATGACACGCATACTG	I-PCR primer
Wbd-1	GCGC *TCTAGA*GGGGCTTCCTGTTTGATGCC *Xba *I	Coding region primer
Wbd-2	GCGC *GTCGAC*CCTCAACCAGCAGTAGGAAG *Sal *I	Coding region primer
W-GFP	GCGC *CTCGAG*ATACCACTGACTTGATCCAG *Xho *I	Coding region primer
*AtEF-1α-F*	ATGGGTAAAGAGAAGTTTCACAT	RT-PCR primer
*AtEF-1α-R*	CTTGTTACAACAGCAGATCAT	RT-PCR primer
*AtPR1-F*	GACGAGAGGGTTGCAGCCTATG	RT-PCR primer
*AtPR1-R*	GATTCTCGTAATCTCAGCTCT	RT-PCR primer
*AtPR4-F*	CCACCTACCATTTCTATAATCC	RT-PCR primer
*AtPR4-R*	CACAGTCGAGAAATTGGTAGTC	RT-PCR primer
*AtPDF1.2-F*	TCATGGCTAAGTTTGCTTCC	RT-PCR primer
*AtPDF1.2-R*	AATACACACGATTTAGCACC	RT-PCR primer

Amino acid and nucleotide sequences of other plant MAPK genes were retrieved from GenBank (http://www.ncbi.nlm.nih.gov/Genbank). Amino acid sequence alignments were performed using the DNAman 6.0 program. The phylogenetic tree was constructed by the Neighbour-Joining method using MEGA 4.

### Amplification of the *GhMPK16 *genomic sequence

Total genomic DNA was extracted from cotton seedlings using a modified cetyl-trimethyl-ammonium bromide (CTAB) method [25]. The genomic DNA of cotton was isolated to clone the DNA sequence and the 5'-flanking region of *GhMPK16*.

Two genomic DNA fragments were PCR amplified with primers designed according to the *GhMPK16 *cDNA sequence. The first fragment was amplified with primers FL1 and Z1, and the second fragment was amplified with primers FL2 and Z2.

The promoter region was cloned using the inverse-PCR (I-PCR) method. Five restriction endonucleases (*Bam *I, *Bgl *II, *Dra *I, *Hind *III and *Xba *I) were used to digest the genomic DNA. Using T4 DNA ligase (TaKaRa, Dalian, China), the DNA fragments were self-ligated to form circles that were used as the template to amplify the *GhMPK16 *promoter region. The 5' flanking region of *GhMPK16 *was obtained from the template digested with *Dra *I. The exterior primers Wup1 and Wdra1 and the interior primers Wup2 and Wdra2 were used for the first and second rounds of PCR, respectively. The sequences of the primers used in this study are provided in Table 1. The PlantCARE program (http://bioinformatics.psb.ugent.be/webtools/plantcare/html) was used to analyze the *GhMPK16 *promoter sequences, which used default parameters.

### Subcellular localization and Histochemical Analysis

To observe the cellular localization of GhMPK16, the GhMPK16::GFP fusion construct was prepared. The *GhMPK16 *coding region was amplified with primers Wbd-1 and W-GFP, containing an *Xba *I site upstream and an *Xho *I site downstream of the deleted stop codon, respectively. The resulting fragment was inserted into the *Xba *I/*Xho *I site of the binary vector pBI121-GFP, and the fragment was fused to GFP and placed under the control of the cauliflower mosaic virus (CaMV) 35S promoter. Particle bombardment was performed according to the manufacturer's instructions (PDS-1000, Bio-Rad Laboratories, Hercules, CA), using gold particles (1.0 μl) and 1350 psi helium pressure. After bombardment, tissues were incubated on MS agar medium under dark conditions at 23°C for 12 h. Nuclei were stained with 100 μg/ml of 4',6-diamidino-2-phenylindole (DAPI) (Solarbio, Beijing, China) in phosphate-buffered saline for 10 min. GFP fluorescence and DAPI fluorescence were examined with a laser scanning microscope (LSM 510 META, ZEISS, Germany) using excitation wavelengths of 488 nm and 350 nm, respectively.

### Immunoblot analysis

The GhMPK16::GFP recombinant plasmid was introduced into the *Agrobacterium tumefaciens *strain GV3101. The *A. tumefaciens *strains were grown overnight in YEB media. Pellet and resuspended cells was performed as described by Guo [26]. Resuspended cells were infiltrated into leaves of 4-week-old *Nicotiana benthamiana *plants as described previously [27,28]. Epidermal cell layers of plant leaves were assayed for fluorescence with a fluorescence microscope (BX51, model 7.3; Olympus) 3 d after injection. Total plant proteins were extracted as Silverstone described [29]. The proteins were separated by sodium dodecyl sulphate-polyacrylamide gel electrophoresis (SDS-PAGE) (12%) and analyzed on immunoblots using a 1000-fold dilution of anti-GFP polyclonal antibodies (Beyotime, Haimen, China) and a 5000-fold dilution of horseradish peroxidase-conjugated goat anti-rabbit IgG (Beyotime). The signals were detected by chemiluminescence.

### Southern blot hybridisation

Genomic DNA (30 μg/sample) was digested with *Eco*R V, *Eco*R I, *Xba *I or *Hin*d III then separated on 0.8% agarose gels by electrophoresis and transferred onto a Hybond-N^+ ^Nylon membrane (Amersham, Pharmacia, UK). A gene-specific dCTP-[α-^32^P]-labelled probe was synthesised with the *GhMPK16 *fragment (primers FL1 and 5P2) using the Primer-a-Gene^® ^Labeling System (Promega, Madison, WI, USA) according to the manufacturer's instruction. The hybridisation was carried out for 24 h at 42°C. After the hybridisation, the blots were washed two times with 2 × SSC, 0.1% SDS and three times with 0.2 × SSC, 0.1% SDS for 10 min each at 42°C, and the radioactive signal was visualised using a FLA-7000 phosphorimager (FUJIFILM).

### RNA blot hybridisation and semi-quantitative RT-PCR analyzes

Total RNA was isolated from cotton seedling leaves using the method described above. Total RNA (15 μg) from each sample was separated on a 1.0% agarose-formaldehyde gel and transferred to Hybond-N^+ ^Nylon membranes (Amersham, Pharmacia, UK). Gene-specific dCTP-[α-^32^P]-labelled probe was synthesis (primers FL2 and 5P3) and RNA blot hybridisation were performed using the methods described for the Southern blot hybridisation.

To analyze gene expression in transgenic plants by RT-PCR, total RNA was isolated from wild-type and transgenic plants using the method described above for gene cloning. Amplifications were performed at 94°C for 5 min, followed by 25-30 cycles of amplification (94°C for 50 s, 50°C for 50 s, and 72°C for 40 s). The PCR products were separated on a 1.8% agarose gel and visualised after ethidium bromide staining. To ensure that equal cDNA amounts were used in each reaction, *EF-1α *was used as a loading control. The gene-specific primers used in RT-PCR analyzes are listed in Table 1.

### Vector construction and plant transformation

The *GhMPK16 *coding region was amplified using primers Wbd-1 and Wbd-2 (Table 1). The amplified fragment was then subcloned into the binary vector pBI121 under the control of the CaMV 35S promoter. The recombinant plasmid was introduced into the *A. tumefaciens *strain GV3101. *Arabidopsis *was transformed using the floral dip method [30]. Transformants were selected on 1/2 MS agar medium containing 30 μg/ml of kanamycin. The transgenic T_3 _lines were used in the experiments.

### Histochemical detection of H_2_O_2_

The substrate, 3,3'-diaminobenzidine (DAB), was used to visually detect H_2_O_2 _in plants [31]. *Arabidopsis *plants (one-week-old) were treated with 200 mM mannitol for 0, 1, 3, and 6 h. The samples were incubated in 1 mg/ml DAB solution (pH 3.8) for 6 h, and then treated with 95% ethanol to remove chlorophyll. In the presence of H_2_O_2_, DAB is locally polymerised and produces visible brown stain.

## Authors' contributions

JS carried out most of the experiments, and drafted the manuscript. LZ participated in southern blot analysis, RNA blot analysis and subcellular localization analysis of the study. HA, CW and XG conceived the experimental design and helped to draft the manuscript. All authors read and approved the final manuscript.

## Supplementary Material

Additional file 1 **Figure S1 **Schematic representation of the genome structure of *GhMPK16*. Length of the exons and introns (A) *Arabidopsis thaliana *(*AtMPK16*), (B) *Gossypium hirsutum *(*GhMPK16*), (C) *Oryza sativa *(Os11g0271100), and (D) *Vitis vinifera *(Vitis vinifera hypothetical protein LOC100246022) are indicated according to the scale below. The exons and introns are highlighted with gray and white bars, respectively. The start codons (ATG) are indicated by (▼), and the stop codons are marked by (*). Click here for file

Additional file 2 **Figure S2**. Analysis of the integrity of GhMPK16::GFP fusion protein. (A) Transient expression of *35S-GFP *and *35S-GhMPK16::GFP *in *N. benthamiana *cells. Bar = 10 μm. (B) Immunoblot analysis of GhMPK16::GFP fusion protein. 4-week-old *N. benthamiana *plants were chosen, and proteins were isolated from wild-type and transgenic plant leaves. Each lane was loaded with a total of 50 μg protein. Click here for file
