# Supplementary figures and images for: GhMPK16, a novel stress-responsive group D MAPK gene from cotton, is involved in disease resistance and drought sensitivity
Source: BMC Mol Biol. 2011 May 16;12:22. doi: 10.1186/1471-2199-12-22 (PMC3117701; doi:10.1186/1471-2199-12-22)

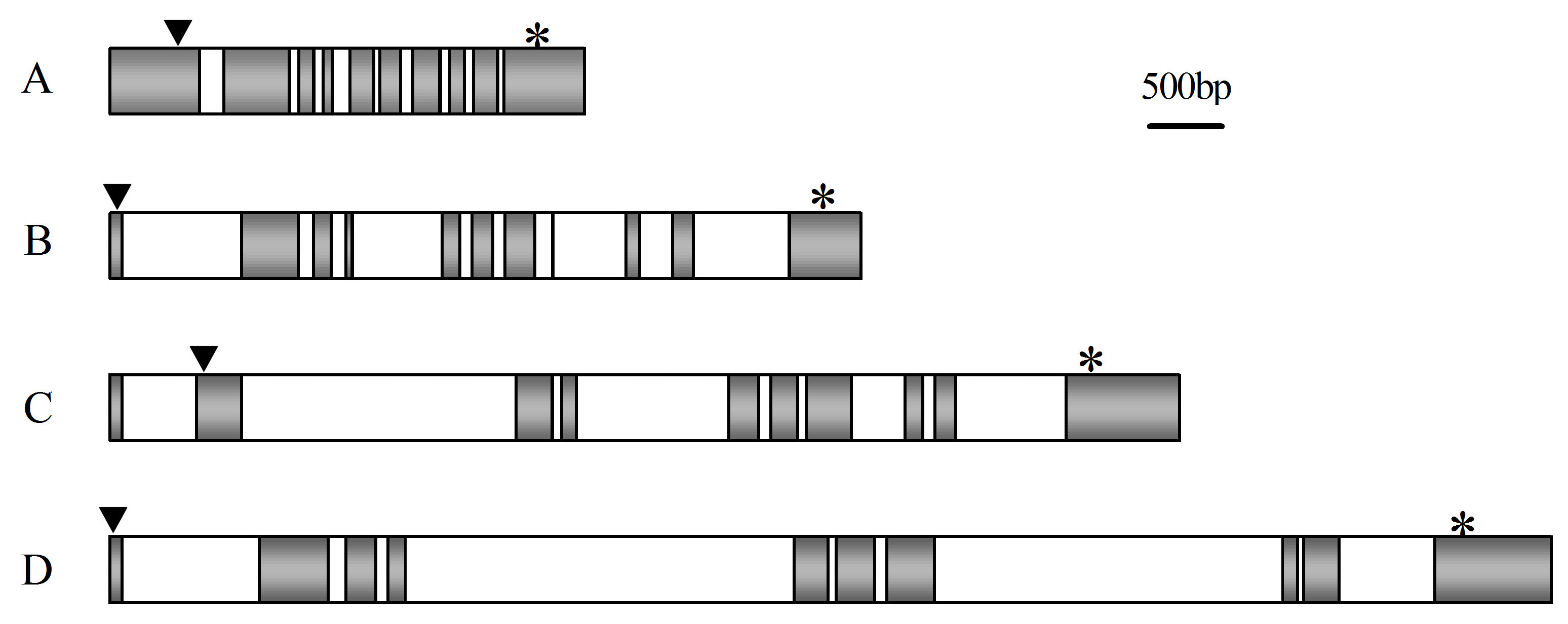

Supplement: Additional file 1 — Figure S1 Schematic representation of the genome structure of GhMPK16. Length of the exons and introns (A) Arabidopsis thaliana (AtMPK16), (B) Gossypium hirsutum (GhMPK16), (C) Oryza sativa (Os11g0271100), and (D) Vitis vinifera (Vitis vinifera hypothetical protein LOC100246022) are indicated according to the scale below. The exons and introns are highlighted with gray and white bars, respectively. The start codons (ATG) are indicated by (▼), and the stop codons are marked by (*). [file 1471-2199-12-22-S1.JPEG]

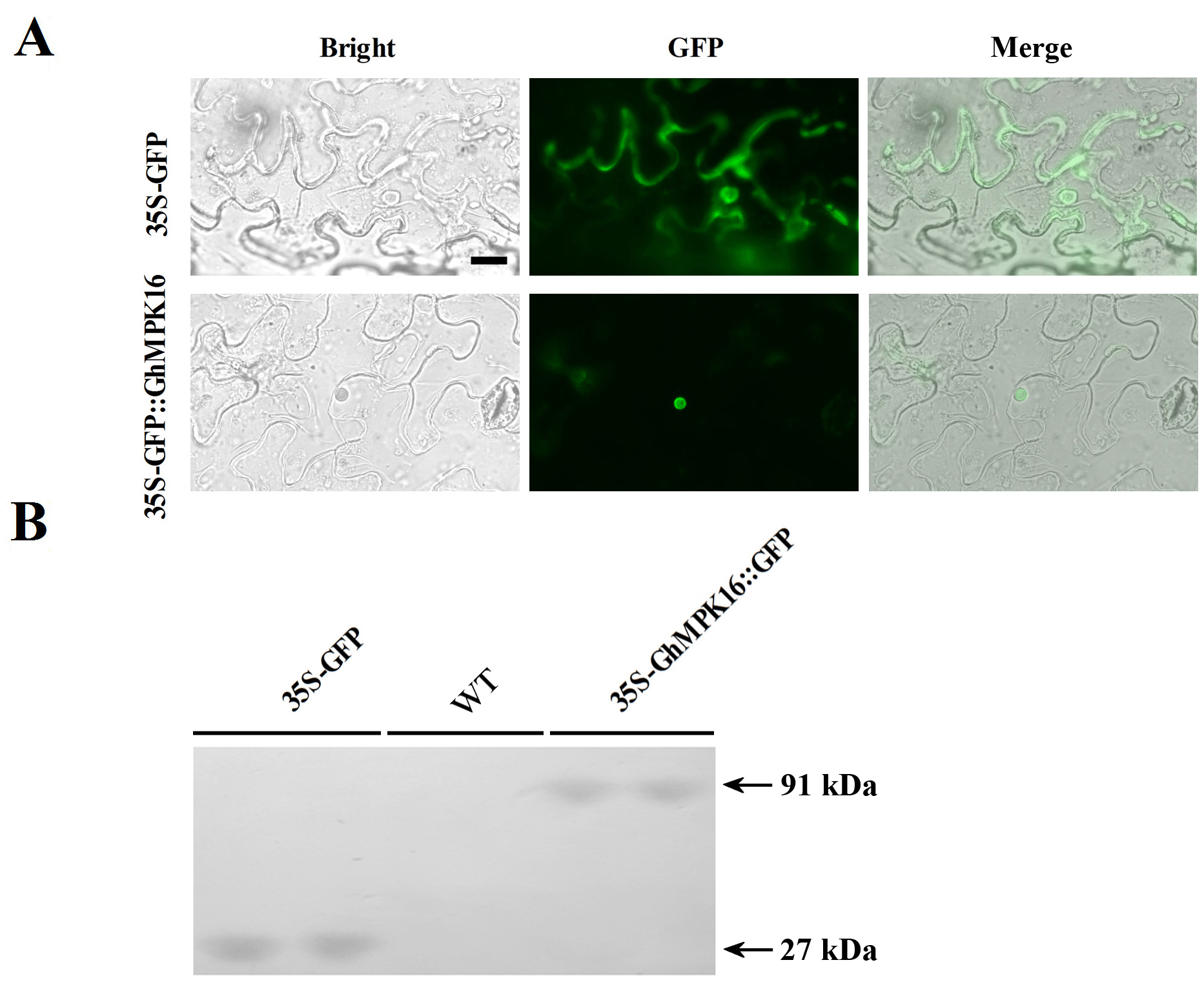

Supplement: Additional file 2 — Figure S2. Analysis of the integrity of GhMPK16::GFP fusion protein. (A) Transient expression of 35S-GFP and 35S-GhMPK16::GFP in N. benthamiana cells. Bar = 10 μm. (B) Immunoblot analysis of GhMPK16::GFP fusion protein. 4-week-old N. benthamiana plants were chosen, and proteins were isolated from wild-type and transgenic plant leaves. Each lane was loaded with a total of 50 μg protein. [file 1471-2199-12-22-S2.JPEG]
